# Deregulation of stemness and senescence genes in bone marrow mesenchymal stem cells of multiple myeloma: implications for therapeutic approaches

**DOI:** 10.1007/s44313-026-00128-3

**Published:** 2026-05-07

**Authors:** Fatemeh Soleymani, Saeideh Kavousi, Nastaran Khodakarim, Mohammad Ahmadvand

**Affiliations:** 1 Department of Medical Genetics, Faculty of Medical Sciences, Tarbiat Modares University, Tehran, Iran; 2 Department of Medical Oncology and Hematology, Iran University, Tehran, Iran; 3 Cell Therapy and Hematopoietic Stem Cell Transplantation Research Center, Research Institute for Oncology, Hematology and Cell Therapy, Tehran University of Medical Sciences, Tehran, Iran

**Keywords:** Multiple Myeloma, Tumor Microenvironment, Bone Marrow Mesenchymal Stem Cells, Stemness, Senescence-Related Genes

## Abstract

**Purpose:**

Multiple myeloma (MM) is a hematologic malignancy associated with a poor prognosis. MM-derived mesenchymal stromal cells (MM-MSCs) contribute to disease progression by creating a supportive stromal microenvironment for malignant cells. Elucidating transcriptomic alterations in MSCs may therefore facilitate the development of novel therapeutic strategies for treatment-resistant MM.

**Methods:**

Total RNA was extracted from cultured MSCs isolated from bone marrow aspirates of patients with MM and normal donors (ND-MSCs). Expression of stemness markers (*NANOG*, *OCT4*) and senescence-associated genes (*P16*, *P21*, *IL-6*, *IL-8*) was analyzed using reverse transcription-quantitative polymerase chain reaction. Cellular senescence was assessed by senescence-associated β-galactosidase (SA-β-gal) staining.

**Results:**

Compared with ND-MSCs, MM-MSCs demonstrated a higher percentage of SA-β-gal-positive cells. Gene expression analysis showed upregulation of *P21* and *IL-6* in MM-MSCs, whereas *NANOG* and *OCT4* were significantly downregulated. Notably, this downregulation was consistent with analysis of a publicly available RNA sequencing dataset, supporting the validity of our findings.

**Conclusions:**

Consistent with previous reports of increased senescence, our findings demonstrate significant downregulation of stemness-related genes (*NANOG*, *OCT4*) in MSCs from newly diagnosed, untreated patients with MM. This concurrent dysregulation of stemness and increased senescent phenotype may contribute to the pathogenicity of the tumor microenvironment and represents a potential target for therapeutic intervention.

**Clinical Trial Registration:**

Not applicable. This study did not involve a clinical trial.

**Graphical Abstract:**

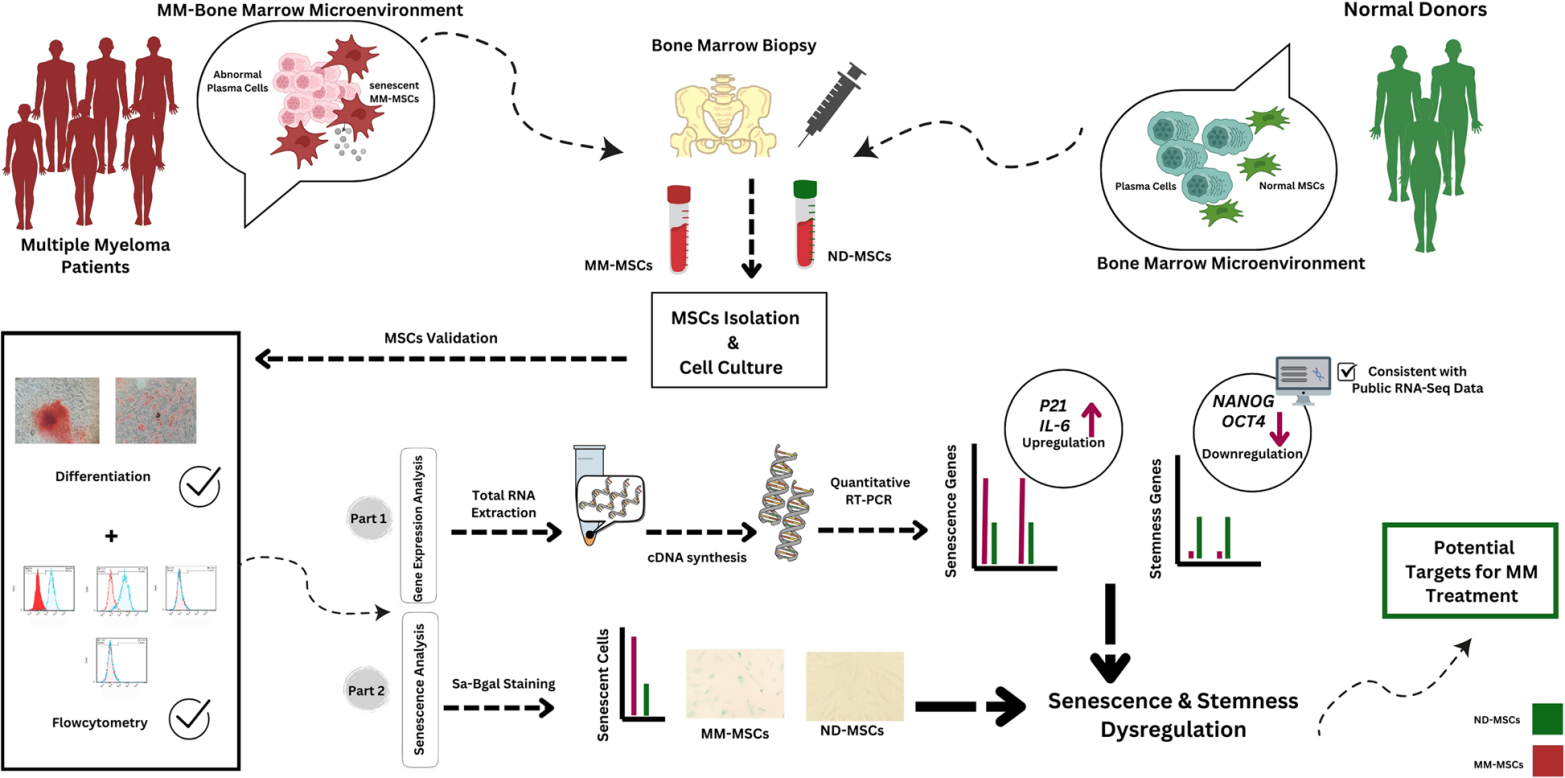

**Supplementary Information:**

The online version contains supplementary material available at 10.1007/s44313-026-00128-3.

## Introduction

Multiple myeloma (MM) is a common hematologic malignancy, with an estimated 36,110 new cases (representing 1.8% of all new cancer cases) and 12,030 associated deaths (accounting for 1.9% of all cancer deaths) in the United States in 2025 [1]. Despite substantial therapeutic advances, MM remains largely incurable and is associated with poor long-term prognosis.

MM is characterized by the clonal expansion of malignant plasma cells (PCs) within the bone marrow [2]. Extensive research has explored the role of the bone marrow microenvironment and its intricate interactions with PCs [3]. This microenvironment comprises both cellular and non-cellular components. Cellular compartments include hematopoietic and stromal cells, whereas non-cellular compartments consist of cytokines, growth factors, chemokines, and the extracellular matrix [4]. Among these, mesenchymal stem cells (MSCs) are key constituents that play critical roles in both physiological and pathological bone marrow processes.

Multiple studies have demonstrated that patients with MM have abnormal MSCs with multiple reproducible differentially expressed genes compared to normal mesenchymal stem cells [5, 6]. In particular, these cells can overproduce growth factors such as interleukin-6 (IL-6), which promotes myeloma cell proliferation [7]. Comparative gene expression analyses of MM-derived MSCs (MM-MSCs) and MSCs from normal donors (ND-MSCs) have also revealed downregulation and disruption of genes involved in cell cycle regulation, providing further evidence of dysregulated transcriptional programs in MM-MSCs [5]. Functionally, MM-MSCs are believed to contribute to disease progression by supporting tumor growth, promoting treatment resistance, and establishing a permissive stromal microenvironment [8].

Compared with bone marrow-derived ND-MSCs, MM-MSCs exhibit pronounced features of cellular senescence [9, 10]. These changes have been strongly associated with MM development, driven in part by myeloma cell stimulation through lysophosphatidic acid receptors 1 (LPA1) and 3 (LPA3) [8]. This association is significant because previous studies show that the senescence-associated secretory phenotype and altered metabolic activity of senescent MSCs exert pro-tumorigenic effects, promoting tumor growth and facilitating processes that contribute to MM progression [11].

Cellular senescence is characterized by irreversible cell-cycle arrest in the G0-G1 phase, primarily mediated by cyclin-dependent kinase inhibitors such as P21 and P16. This state is typically associated with increased β-galactosidase activity, overexpression of cytokines, and abnormal cellular morphology [12]. Although the precise mechanisms underlying MSC senescence in MM remain unclear, understanding these pathways is important because they may provide novel therapeutic targets for improving treatment outcomes.

As multipotent adult stem cells, MSCs are defined by their dual capacity for self-renewal and multilineage differentiation into various tissue types [13]. However, accumulating evidence indicates that these fundamental properties—proliferation and differentiation—are impaired in MM. Key transcription factors, including *OCT4* and *NANOG*, regulate these functions in MSCs [14]. Studies show that MM-MSCs display reduced differentiation capacity [15, 16], significantly decreased proliferative potential, and severely impaired osteoblastic differentiation compared with ND-MSCs [6]. The high prevalence of senescent cells in MM-MSCs likely contributes to these functional impairments, as senescence is well known to disrupt normal cellular function.

Interestingly, previous studies provide preliminary evidence that targeting MSC senescence may shift MM-MSCs from a tumor-promoting to a tumor-inhibiting phenotype [8]. Thus, modifying the altered cellular characteristics of MM-MSCs may represent a promising strategy for improving therapeutic outcomes.

Although previous studies have highlighted an increased senescent phenotype in MM-MSCs, the concurrent status of core stemness pathways in these cells remains poorly understood. Based on this knowledge gap, we hypothesized that MSCs from newly diagnosed patients with MM exhibit dual dysregulation characterized by increased senescence and reduced stemness. We therefore compared the expression of key senescence markers (*P16*, *P21*, *IL-6*, *IL-8*) and stemness-associated genes (*NANOG*, *OCT4*) between MM-MSCs and ND-MSCs. Elucidating this potential dual dysregulation is crucial for identifying pathological mechanisms and may ultimately inform targeted strategies to disrupt these interactions, slow disease progression, and improve treatment outcomes.

## Materials and Methods

### Patients

This study included patients referred to Rasoul Hospital, Iran University of Medical Sciences, Tehran, Iran. Bone marrow biopsies were initially performed in a cohort of 10 patients for MM diagnosis. The final study cohort comprised six patients who met the inclusion and exclusion criteria. Additionally, three normal bone marrow biopsy samples were collected for comparison. Table 1 summarizes the demographic and clinical characteristics of the study participants. Importantly, there were no statistically significant differences between the patient and normal donor groups in age (*p* = 0.19) or sex distribution (*p* > 0.99). These samples were used to examine the molecular characteristics of MM-MSCs and ND-MSCs.

**Table 1:** Demographic and clinical characteristics of patients with multiple myeloma and normal donors

**Characteristic**	**MM Patients (*n*=6)**	**Normal Donors (*n*=3)**	***p*-value**
***age***			0.19
Mean ± SD	61.7 ± 9.87	55.3 ± 2.52	
Range (Min–Max)	48–77	53–58	
***Gender***			>0.99
Male	3	2	
Female	3	1	
***ISS Stage***			NA
Stage II	4	NA	
Stage III	2	NA	
Key Comorbidities*	None	None	NA

*p*-values were calculated using the Mann–Whitney *U* test for age and Fisher’s Exact Test for sex. *Abbreviations:* *MM* Multiple Myeloma, *NA* Not Applicable

^*^Key comorbidities were defined based on the study's exclusion criteria as any active or prior malignancy, or receipt of active treatment for diabetes mellitus or hypothyroidism; consequently, none of the enrolled participants presented with these conditions.

### Inclusion and Exclusion Criteria

Patients with a confirmed MM diagnosis at intermediate to advanced disease stages (Stage II/III) were eligible for enrollment*.* Disease staging was determined according to the International Staging System (ISS). ISS classification was based on serum β2-microglobulin (β2M) and serum albumin (ALB) levels as follows: Stage I, β2M < 3.5 mg/L and ALB ≥ 3.5 g/dL; Stage II, β2M < 3.5 mg/L and ALB < 3.5 g/dL, or 3.5 mg/L ≤ β2M < 5.5 mg/L; and Stage III, β2M ≥ 5.5 mg/L.

Exclusion criteria included the presence of systemic disorders (such as inflammatory conditions, genetic diseases, and multiple sclerosis, prior treatment for MM, a history of other malignancies, or treatment for other systemic conditions, including hypothyroidism or diabetes mellitus. Healthy individuals without a history of systemic disease, inflammation, or ongoing systemic therapy were recruited as normal bone marrow donors for transplantation.

### MSC Isolation and Culture

A bone marrow aspiration was performed according to standard clinical procedures. Approximately 3–5 mL of bone marrow aspirate was obtained from the posterior iliac crest. MSCs were isolated using the plastic adherence method and cultured in low-glucose medium *(*bioIDEA, Tehran, Iran) supplemented with 10% fetal bovine serum (FBS; bioIDEA) and 1% penicillin/streptomycin (bioIDEA). Cells were maintained at 37 °C in a humidified atmosphere containing 5% CO_2_ until reaching approximately 80% confluence. They were then detached using 0.25% trypsin/ethylenediaminetetraacetic acid (bioIDEA), centrifuged at 1,200 rpm for 5 minutes, and subsequently passaged. This protocol was applied to both MM-MSCs and ND-MSCs. All isolated MSC populations successfully reached passage 4 for subsequent analyses.

### MSC Validation

To validate MSCs, several established criteria must be satisfied, including spindle-shaped morphology, adherence to plastic surfaces, the capacity to differentiate into at least two cell lineages, and the expression or absence of specific clusters of differentiation (CD) markers. MSCs were cultured in osteogenic and adipogenic differentiation media (bioIDEA), and differentiation was confirmed according to the manufacturer’s protocol. Briefly, MSCs were seeded in 12-well plates and maintained in lineage-specific differentiation media, which were replaced every 3 days. After 3 weeks, cells designated for osteogenic assessment were fixed with 4% formaldehyde and stained with 4% Alizarin Red. For adipogenic evaluation, cells were fixed with 10% formalin and stained with 1% Oil Red (bioIDEA).

In addition to differentiation capacity, harvested cells were analyzed by flow cytometry to confirm MSC immunophenotypes, characterized by the expression of CD105 and CD90, and the absence of CD45, CD34, and human leukocyte antigen–DR (HLA-DR). Briefly, cells were detached using 0.25% trypsin (bioIDEA), and the resulting pellet was resuspended in 5% FBS. Cells were then incubated with monoclonal antibodies in the dark for 30 minutes. Marker expression was assessed using a FACSCalibur™ flow cytometer (Becton Dickinson, USA), and data were analyzed with FlowJo version 10 to confirm MSC phenotype.

### RNA Extraction and cDNA Synthesis

Total RNA was extracted from MSCs (1 × 10^6^ cells) using TRIzol reagent (Sigma, Germany) according to the manufacturer's instructions. RNA purity and concentration were assessed using a NanoDrop spectrophotometer (IMPLEN GmbH, Munich, Germany). Acceptable quality was confirmed by absorbance ratios of approximately 2.0 at 260/280 nm and ≥1.8 at 260/230 nm. For cDNA synthesis, 10 µl of extracted RNA was used for first-strand synthesis with the PrimeScript cDNA Synthesis Kit (Takara Bio, Japan), following the manufacturer's instructions.

### Quantitative RT‐PCR for Gene Expression Level Evaluations

As previously reported [17, 18], Target gene expression was quantified by reverse transcription-quantitative polymerase chain reaction (RT-qPCR) using a StepOne ABI system. Each reaction was performed with high ROX RealQ Plus 2x Master Mix (Amplicon, Denmark), according to the manufacturer’s instructions. Glyceraldehyde-3-phosphate dehydrogenase (*GAPDH*) served as the endogenous reference gene for normalization. All primers were synthesized by Sinaclon Co. (Tehran, Iran), and their sequences are provided in Online Resource 1.

### Statistical analysis

Demographic characteristics (age and sex) were compared between the MM-MSC and ND-MSC groups using the Mann–Whitney *U* test and Fisher's exact test, respectively. The Mann–Whitney *U* test, a nonparametric statistical method, was also used to compare expression levels of the studied genes between MM-MSCs and ND-MSCs. Each sample was analyzed in triplicate, and the mean cycle threshold (Ct) value was used for quantification. Relative gene expression was quantified using the comparative ΔΔCt method. Specifically, Ct values of target genes were normalized to the endogenous control, *GAPDH*, to obtain ΔCt values (ΔCt = Ct*target gene* − Ct*GAPDH* ​). The mean ΔCt of the ND-MSC group served as the reference. Final statistical comparisons were conducted using ΔCt values between groups. Data analysis was performed using GraphPad Prism 9 (GraphPad Software Inc., San Diego, CA, USA). A two-tailed *p*-value <0.05 was considered statistically significant.

### Public dataset analysis

Publicly available RNA sequencing (RNA-seq) data from the Gene Expression Omnibus (GEO) dataset GSE196297 [19] were analyzed to validate the stemness-related gene expression patterns observed in our study. This dataset includes bulk RNA-seq counts from bone marrow-derived MSCs obtained from patients with MM and healthy control donors. In the original study, RNA-seq data were processed using DESeq2 for normalization and differential expression analysis. Gene Set Enrichment Analysis was performed using MSigDB hallmark gene sets. The present analysis utilized the DESeq2-normalized count data provided by the original study. To ensure consistency with our patient group, only patients with stage II/III MM were included and compared with control donors. Log2-transformed expression levels of*POU5F1* (*OCT4*) and *NANOG* were compared between patients with MM (n = 4) and control donors (n = 5) using the Mann–Whitney *U* test.

### SA-β-gal Staining (X-GAL)

Senescence-associated β-galactosidase (SA-β-gal) staining was performed to determine the proportion of senescent cells in MM-MSCs and ND-MSCs. Cells were seeded in 12-well plates and cultured to achieve approximately 80% confluence. Staining followed the protocol established by Debacq-Chainiaux et al. [20].

Briefly, cells were washed with phosphate-buffered saline (PBS) and fixed in freshly prepared solution containing 2% formaldehyde (vol/vol) and 0.2% glutaraldehyde (vol/vol) in PBS. After two additional PBS washes, cells were incubated overnight at 37 °C without CO_2_ in a fresh staining solution consisting of 40mM citric acid/sodium phosphate buffer (pH 6), 5mM potassium ferricyanide, 5mM potassium ferrocyanide, 2mM magnesium chloride, 150mM sodium chloride, and 1 mg/mL 5-bromo-4-chloro-3-indolyl-β-D-galactosidase (X-gal in distilled water. Following approximately 16 hours of incubation, cells were washed thrice with PBS, rinsed once with methanol, and air-dried. Senescent cells were quantified by counting at least 500 cells across 5–10 randomly selected, non-overlapping fields per sample. The percentage of senescence was calculated as the proportion of SA-β-gal-positive (blue) cells relative to the total number of cells counted. To ensure inter-observer consistency, quantification was performed independently by two observers; however, the assessment was not conducted in a blinded manner.

## Results

### Identification and Differentiation of MSCs

**Fig. 1:**
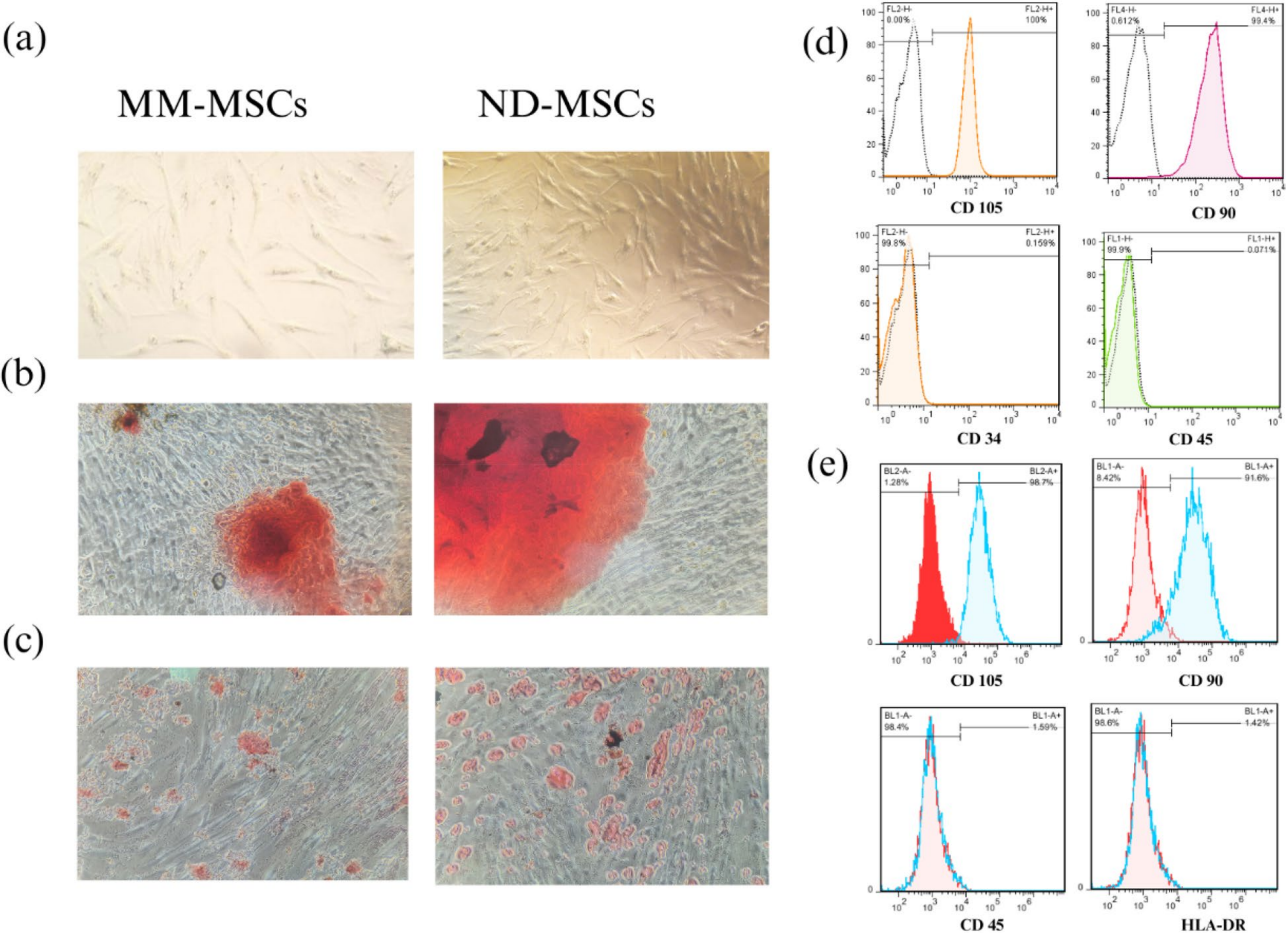
Isolation and characterization of MSCs from ND and MM patients. **a** Representative micrographs showing the typical spindle-shaped morphology of MM-MSCs (left) and ND-MSCs(right). **b** Osteogenic differentiation was confirmed by Alizarin Red staining of calcium deposits. **c** Adipogenic differentiation was verified by Oil Red O staining of intracellular lipid droplets. (original magnification: ×100) (**d**–**e**) Flow cytometry analysis confirmed the immunophenotype of MSCs. **d** ND-MSCs were positive for CD105 and CD90, and negative for CD45 and CD34. **e** MM-MSCs were positive for CD105 and CD90, and negative for CD45 and HLA-DR. CD, cluster of differentiation; HLA-DR, human leukocyte antigen DR; MSC, mesenchymal stem cell; MM multiple myeloma; ND, normal donor

MSCs isolated from bone marrow exhibited a spindle-shaped morphology (Fig. 1a) and demonstrated multipotent differentiation capacity. Osteogenic differentiation was confirmed by the presence of mineralized extracellular matrix deposits following Alizarin Red staining (Fig. 1b). Adipogenic differentiation was verified by the presence of lipid droplet formation after Oil Red O staining (Fig. 1c). Flow cytometry analysis showed positive expression of CD105 and CD90 and absence of CD45. ND-MSCs were negative for CD34 (Fig. 1d), whereas MM-MSCs were negative for HLA-DR (Fig. 1e).

### Senescence-Related Genes Demonstrating Abnormal Expression Patterns in MM-MSCs

**Fig. 2:**
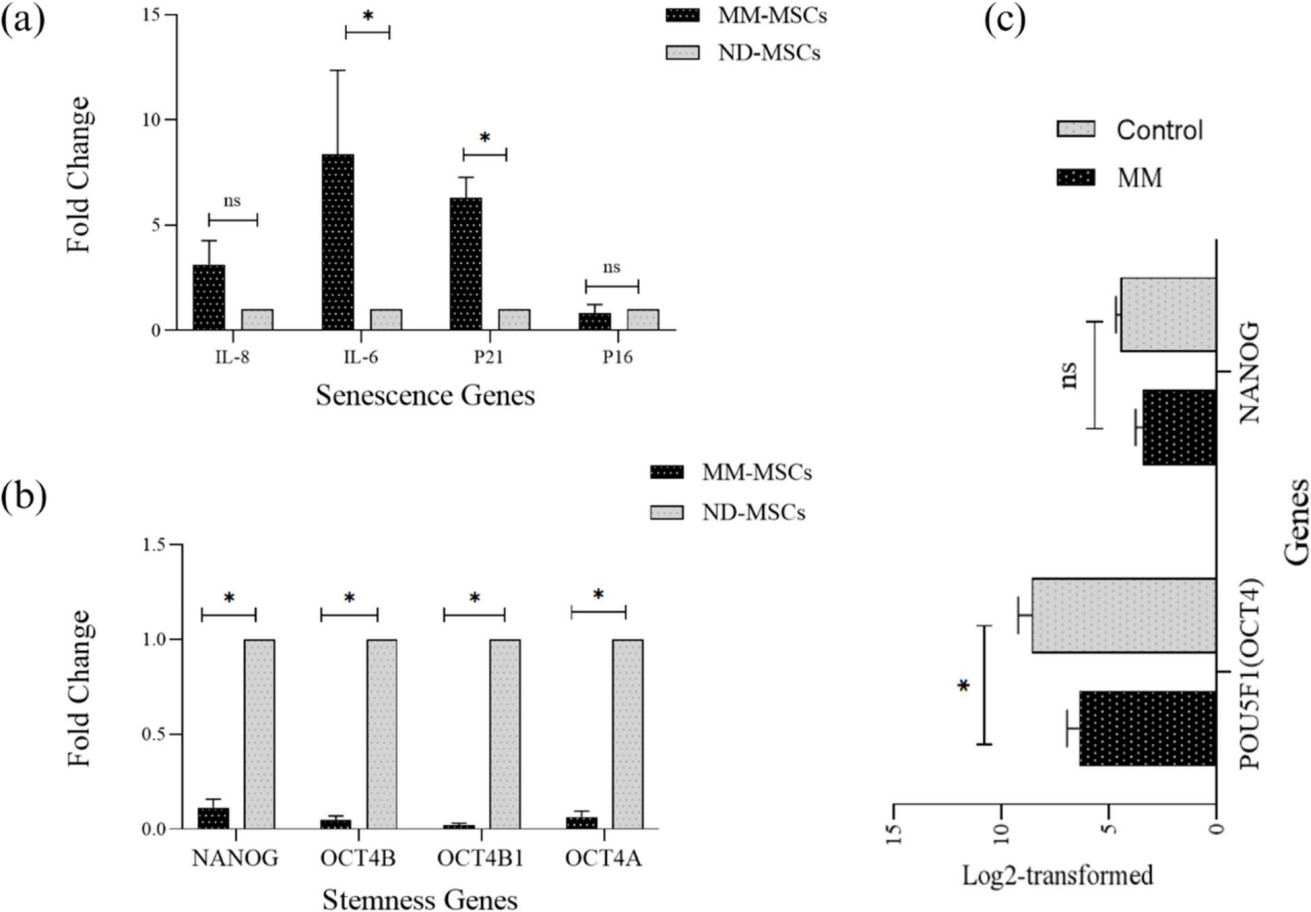
Dysregulation of senescence- and stemness-related gene expression in MM-MSCs. **a** Relative expression levels of senescence-related genes (*P16*, *P21*, *IL-6*, *IL-8*) in MM-MSCs compared to ND-MSCs. **b** Relative expression of stemness-related genes *(NANOG, OCT4)* between MM-MSCs (*n*=6) and ND-MSCs (*n*=3). **c** Validation using public RNA sequencing data (GSE196297), showing Log2-transformed normalized counts for *POU5F1* (*OCT4*) and *NANOG* in patients with MM (*n*=4) and control donors (*n*=5). All data are presented as mean ± SEM. The significance level is indicated as follows: ns, not significant; ^**∗**^*p* ≤ 0.05. MSC, mesenchymal stem cell; MM multiple myeloma; ND, normal donor

*IL-6*, a pro-inflammatory cytokine gene, was significantly overexpressed in MM-MSCs compared with ND-MSCs (*p* ≤ 0.05). Additionally, the cell cycle-regulating gene *P21* was significantly upregulated in MM-MSCs (*p* ≤ 0.05), indicating a potential association between cellular senescence and the MM-MSC phenotype. In contrast, no statistically significant differences were observed in the expression of *P16* and *IL-8* between the two groups (Fig. 2a).

### Stemness-Associated Genes are Suppressed in MM-MSCs

MM-MSCs exhibited dysregulation of stemness-associated genes relative to ND-MSCs, with *NANOG* and *OCT4* transcripts (*OCT4A*, *OCT4B*, *OCT4B1*) being significantly differentially expressed in MM-MSCs. All transcripts of *OCT4A* (*p* < 0.05), *OCT4B* (*p* < 0.05), *OCT4B1* (*p* < 0.05), and *NANOG* (*p* < 0.05) were downregulated in MM-MSCs (Fig. 2b).

### Concordance of OCT4 and NANOG Expression in RNA-seq Data

RNA-seq data showed concordance for *OCT4* and *NANOG* expression. Log2-transformed, normalized counts revealed significant downregulation of *POU5F1* (*OCT4*) in patients with MM (*p* = 0.0317) and marginally significant downregulation of *NANOG* (*p* = 0.0635) compared with healthy controls. These findings are consistent with our in-house observations of suppressed stemness gene expression in MM-MSCs (Fig. 2c).

### MM-MSCs Exhibit Increased SA-β-Gal Positivity

**Fig. 3:**
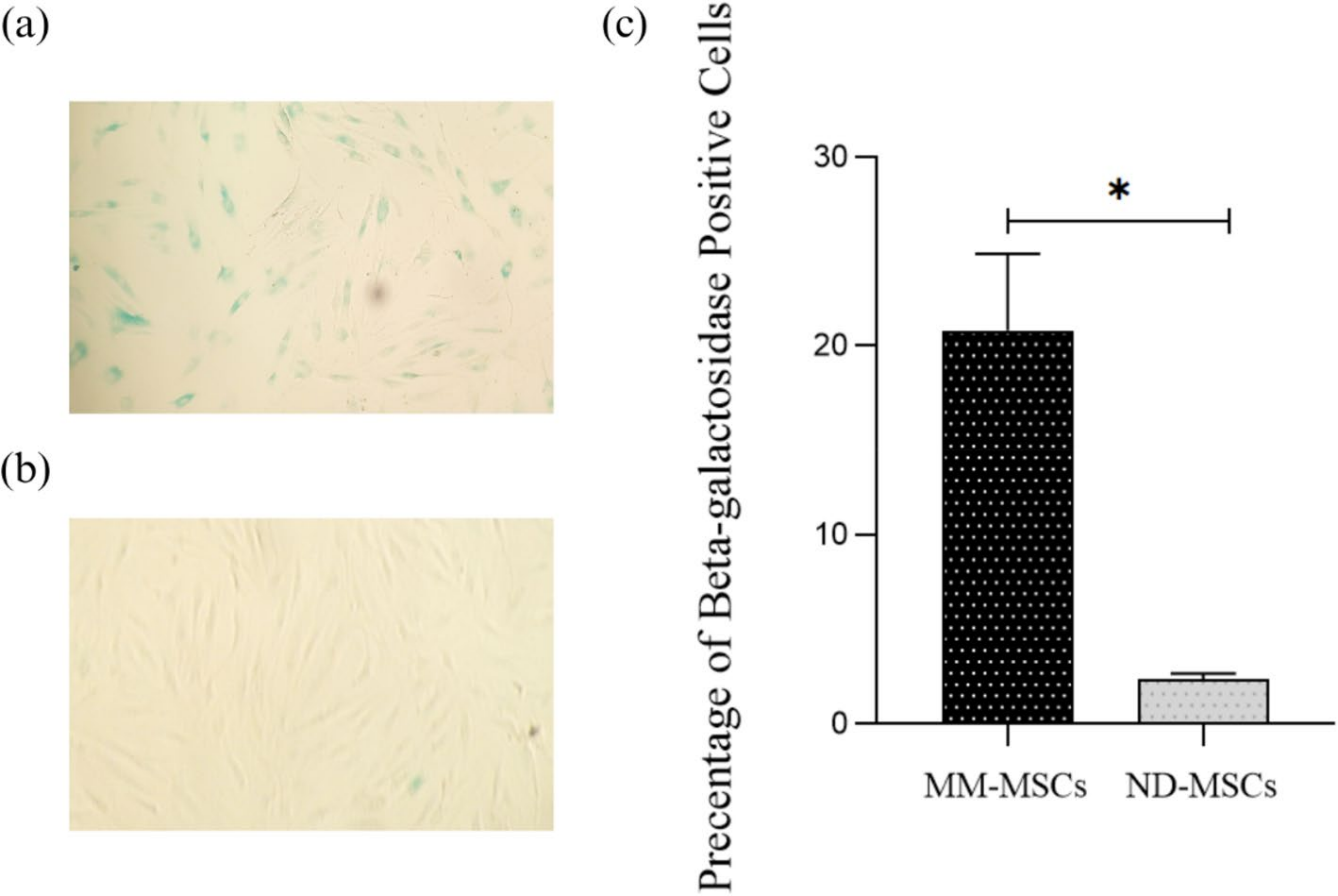
Detection of cellular senescence by SA-β-Gal staining. **a** Representative image showing SA-β-gal staining of MM-MSCs. **b** Representative image showing SA-β-gal staining of ND-MSCs. Senescent cells appear blue (original magnification: ×100). **c** Quantitative analysis of senescent cells. Percentages were calculated by counting blue-stained and total cells across multiple random fields for each sample. MM-MSCs exhibited a significantly higher proportion of senescent cells compared with ND-MSCs. Data in (**c**) are presented as mean ± SEM. ∗: *p* ≤ 0.05. MSC, mesenchymal stem cell; MM multiple myeloma; ND, normal donor; SA-β-Gal, senescence-associated β-galactosidase; SEM, standard error of the mean

SA-β-gal staining revealed a significant difference between MM-MSCs and ND-MSCs. As Fig. 3a–b shows, MM-MSCs displayed a significantly higher proportion of SA-β-gal–positive cells (*p* ≤ 0.0169) (Fig. 3 c).

## Discussion

Our findings indicate that MSCs derived from newly diagnosed, untreated patients with MM exhibit dual dysregulation, characterized by accelerated senescence and a significant loss of stemness. This finding supports the hypothesis that the concurrent disruption of these pathways contributes to MM pathogenesis. Reciprocal interactions between PCs and MM-MSCs have emerged as a critical area of investigation. Several studies suggest that PCs play a crucial role in inducing senescence in surrounding MSCs. Interestingly, senescent MSCs, in turn, appear to promote treatment resistance in MM. Elucidating the mechanisms underlying this bidirectional interaction may inform the development of therapeutic strategies specifically targeting senescence-driven treatment resistance.

Consistent with the dual-dysregulation hypothesis, we observed significant upregulation of the pro-senescent factors *IL-6* and *P21* in MM-MSCs, both established markers of cellular aging and cell cycle arrest. Concurrently, we detected significant downregulation of the core stemness transcription factors *OCT4* and *NANOG*. This concurrent phenotype suggests a profound functional shift in MM-MSCs, whereby cells not only enter a senescent state but also lose their intrinsic self-renewal capacity.

Regarding the senescence aspect, *IL-6*, a key growth factor and marker of senescence, exhibited a significant increase in MM-MSCs, consistent with previous findings [7, 9]. IL-6 plays a pivotal role in sustaining myeloma cell survival and proliferation by activating critical signaling pathways such as JAK/STAT, PI3K/AKT, and MAPK. Previous studies have demonstrated that *IL-6* overexpression in senescent cells not only supports but also perpetuates the proliferation of neighboring malignant cells [21]. These findings further support the conclusion that MM-MSCs exist in a senescent state, aligning with prior studies that underscore their role in promoting tumorigenesis, conferring drug resistance, and sustaining the survival of malignant PCs.

*P16* and *P21* are widely used biomarkers of cellular senescence because their overexpression induces cell cycle arrest. Our findings of elevated P21 expression alongside unchanged P16 levels align with previous reports [9]. This pattern suggests that the role of *P16* in senescence may be context-dependent, as *P16* regulates specific senescent states, while *P53* governs others based on cellular responses to different stressors [22]. Additionally, endogenous P16 has been shown to inhibit *IL-6* expression [23], while IL-6-mediated inhibition of G0-G1 cell cycle progression is mediated through the P21 pathway [24]. Together, these relationships provide a coherent molecular framework that supports our observation of increased *P21* and *IL-6* expression. Although IL-8 is recognized as a senescence-associated cytokine [25], our study only detected a trend towards upregulation that did not achieve statistical significance, likely attributable to the small sample size.

The transcription factors *OCT4* and *NANOG* are critical for the survival and self-renewal of MSCs [26]. *OCT4* establishes a regulatory network that inhibits differentiation-associated genes, thereby preserving pluripotency [27]. Similarly, *NANOG* is crucial for sustaining self-renewal and facilitating pluripotent or multipotent differentiation, while also suppressing spontaneous senescence [28]. Consistent with these findings, we observed significant downregulation of *OCT4* and *NANOG* expression in MM-MSCs compared with ND-MSCs. Although these genes have been identified as key components of the regulatory network that represses differentiation and maintains MSC pluripotency [29], their reduced expression in MM-MSCs appears to be a novel aspect of MM pathophysiology observed in our cohort. Analysis of publicly available RNA-sequencing data from the GEO dataset [19], yielded results concordant with our RT-qPCR findings, further supporting the downregulation of critical stemness genes in MM-MSCs. However, a recent study reported no significant differences in the expression of stemness-related genes between HD-MSCs and MM-MSCs [6]. Notably, Lu et al. investigated primarily treated patients with MM, whereas our study focused on newly diagnosed, untreated patients with MM at intermediate to advanced disease stages. This fundamental difference in patient characterization may account for the discrepancy in gene expression and suggests that disease stage and treatment status could be critical determinants of MSC gene expression. Overall, these results highlight a potential role for *OCT4* and *NANOG* dysregulation in the early stages of MM pathogenesis.

Crucially, the observed loss of stemness and increased senescence in MM-MSCs are not isolated events but likely represent two aspects of a single underlying process, forming the basis for our "dual dysregulation" hypothesis. Our findings support this link by demonstrating concurrent downregulation of *OCT4* and *NANOG,* alongside upregulation of the cell-cycle inhibitor *P21.* This pattern is consistent with established mechanistic evidence: *NANOG* has been shown to counteract senescence [30] and delay its onset through specific signaling pathways [31], while *OCT4* maintains stem cell self-renewal and suppresses senescence partly by inhibiting *P21* expression [32]. Therefore, the simultaneous downregulation of *OCT4* and upregulation of *P21* observed in our study corroborates previous reports describing a negative regulatory axis between these factors. Collectively, these internally consistent findings reinforce the rationale that targeting dysregulated transcription factors may represent a novel therapeutic approach for overcoming treatment resistance in MM.

### Limitations

Several limitations should be considered before generalizing these findings. First, the sample size was relatively small, which may have limited the statistical power of our results. Second, this observational study relied primarily on gene expression profiling and phenotypic characterization (i.e., senescence-associated β-galactosidase staining); the absence of functional assays to directly validate the biological consequences of stemness loss and senescence represents an important limitation. Third, although cohorts were well matched for key confounders such as age (*p*> 0.05), subtle biological effects from these variables cannot be excluded, reflecting a limitation inherent to observational studies. Future studies with larger cohorts and functional validation are warranted to confirm and extend these observations.

## Conclusion

This study provides further evidence implicating senescence-related dysregulation in MM-MSCs, indicating that these cells exhibit a more pronounced senescent phenotype than ND-MSCs. We also demonstrate a significant downregulation of stemness-related genes in MM-MSCs. This dual dysregulation may contribute to the senescent state of MM-MSCs and play a role in the pathogenicity of the tumor microenvironment.

Taken together, these findings highlight the potential for novel therapeutic strategies that target this dual dysregulation. Specifically, modulation of stemness-associated transcription factors may represent a promising avenue for improving treatment outcomes in MM. Further investigation of the signaling pathways driving this phenotype may reveal additional actionable therapeutic targets.

## Supplementary Information

Supplementary Material 1.

## Data Availability

The datasets generated during and/or analyzed during the current study are available from the corresponding author on reasonable request.

## Abbreviations

ALB: Serum Albumin
β2M: Serum β2-microglobulin
CD: Clusters of Differentiation
GAPDH: Glyceraldehyde-3-Phosphate Dehydrogenase
GEO: Gene Expression Omnibus
ISS: International Staging System
MM: Multiple Myeloma
MSCs: Mesenchymal Stem Cells
ND: Normal Donors
PCs: Plasma Cells
RT-qPCR: Reverse Transcription-Quantitative Polymerase Chain Reaction
