# Supplementary material for: Deregulation of stemness and senescence genes in bone marrow mesenchymal stem cells of multiple myeloma: implications for therapeutic approaches
Source: Blood Res. 2026 May 7;61(1):20. doi: 10.1007/s44313-026-00128-3 (PMC13153312; doi:10.1007/s44313-026-00128-3)
Supplement: Supplementary file 1 — Supplementary Material 1. [file 44313_2026_128_MOESM1_ESM.docx]

**Tile: Deregulation of Stemness and Senescence Genes in Bone Marrow Mesenchymal Stem Cells of Multiple Myeloma: Implications for Therapeutic Approaches**

**Journal Name: *Blood Research***

Fatemeh Soleymani, Saeideh Kavousi, Nastaran Khodakarim and Mohammad Ahmadvand ^*^

* Cell Therapy and Hematopoietic Stem Cell Transplantation Research Center, Research Institute for Oncology, Hematology and Cell Therapy, Tehran University of Medical Sciences, Tehran, Iran [mahmadvand@sina.tums.ac.ir](mailto:mahmadvand@sina.tums.ac.ir)

**Table S1 Sequence of oligonucleotide primers used for qRT-PCR.**

| *Target gene* | *Forward sequence* | *Reverse sequence* |
| --- | --- | --- |
| *IL-6* | GTGTGAAAGCAGCAAAGAGGC | CCAGGCAAGTCTCCTCATTGAA |
| *IL-8* | CTCTGTGTGAAGGTGCAGTTTTG | GGTCCACTCTCAATCACTCTCAG |
| *P21(CDKN1A)* | TCTACCACTCCAAACGCC | CACAAACTGAGACTAAGGCAG |
| *P16* | ATGGAGCCTTCGGCTGACT | GTAACTATTCGGTGCGTTGGG |
| *OCT4B* | CAGGGAATGGGTGAATGAC | AGGCAGAAGACTTGTAAGAAC |
| *OCT4B1* | GGGTTCTATTTGGTGGGTTCC | TCCCTCTCCCTACTCCTCTTA |
| *NANOG* | CCTCTATACTAACATGAGTGTGG | CATGGAGGAAGGAAGGAAGAGGAGA |
| *OCT4A* | CGCAAGCCCTCATTTCAC | CATCACCTCCACCACCTG |
| *GAPDH* | ATGGAGAGTAGTACAACAGCCTC | CATGAGTCCTTCCACGATACC |
